# A Model of Functional Brain Connectivity and Background Noise as a Biomarker for Cognitive Phenotypes: Application to Autism

**DOI:** 10.1371/journal.pone.0061493

**Published:** 2013-04-17

**Authors:** Luis García Domínguez, José Luis Pérez Velázquez, Roberto Fernández Galán

**Affiliations:** 1 Temerty Centre for Therapeutic Brain Intervention, Centre for Addiction and Mental Health, University of Toronto, Toronto, Ontario, Canada; 2 Neuroscience and Mental Health Programme, Brain and Behaviour Centre, Division of Neurology, Hospital for Sick Children; Institute of Medical Science and Department of Paediatrics, University of Toronto, Toronto, Ontario, Canada; 3 Department of Neurosciences, School of Medicine, Case Western Reserve University, Cleveland, Ohio, United States of America; University Of Cambridge, United Kingdom

## Abstract

We present an efficient approach to discriminate between typical and atypical brains from macroscopic neural dynamics recorded as magnetoencephalograms (MEG). Our approach is based on the fact that spontaneous brain activity can be accurately described with stochastic dynamics, as a multivariate Ornstein-Uhlenbeck process (mOUP). By fitting the data to a mOUP we obtain: 1) the functional connectivity matrix, corresponding to the drift operator, and 2) the traces of background stochastic activity (noise) driving the brain. We applied this method to investigate functional connectivity and background noise in juvenile patients (n = 9) with Asperger’s syndrome, a form of autism spectrum disorder (ASD), and compared them to age-matched juvenile control subjects (n = 10). Our analysis reveals significant alterations in both functional brain connectivity and background noise in ASD patients. The dominant connectivity change in ASD relative to control shows enhanced functional excitation from occipital to frontal areas along a parasagittal axis. Background noise in ASD patients is spatially correlated over wide areas, as opposed to control, where areas driven by correlated noise form smaller patches. An analysis of the spatial complexity reveals that it is significantly lower in ASD subjects. Although the detailed physiological mechanisms underlying these alterations cannot be determined from macroscopic brain recordings, we speculate that enhanced occipital-frontal excitation may result from changes in white matter density in ASD, as suggested in previous studies. We also venture that long-range spatial correlations in the background noise may result from less specificity (or more promiscuity) of thalamo-cortical projections. All the calculations involved in our analysis are highly efficient and outperform other algorithms to discriminate typical and atypical brains with a comparable level of accuracy. Altogether our results demonstrate a promising potential of our approach as an efficient biomarker for altered brain dynamics associated with a cognitive phenotype.

## Introduction

There is a current debate in the autism field related to the concept of “disconnection” in the autistic brain that became popular from psychological and neuroimaging evidence. Proposals of disruption of coordinated timing in neuronal activity in autism were advanced [1], along with the possibility of reduced brain synchronization [2], but other suggestions appeared indicating that while the brains of those with autism can be perhaps generally characterized as disconnected, local networks may be more connected [3]. Neuroimaging evidence has traditionally supported the concept of reduced functional connectivity in autism, e.g., under-connectivity has been documented in the baseline resting state of cortical networks [4]. Conversely, evidence indicating enhanced connectivity between brain regions has appeared very recently, suggesting that the “under-connectivity theory of autism” may not suffice to describe the brain coordination dynamics characteristic of this condition. For example, enhanced thalamo-cortical connectivity in high-functioning autism has been reported [5], and stronger connectivity between specific cortical areas at rest has also been noted [6], as well as increased connectivity in striatal regions of children with ASD [7]. These findings emphasize that it may not be a matter of less connectivity in autism, but of a different style of coordination dynamics between specific areas and perhaps also globally.

Most of these studies have relied on metabolic measurements. A complementary approach is the analysis of electroencephalographic signals, which have greater time resolution thus allowing for the study of transient coordination patterns. Indeed, the crucial aspect of these patterns in normal cognition is their transience: widespread long-lasting synchrony is normally associated with unconsciousness or disease [8]. Thus, studies evaluating electroencephalographic or magnetoencephalographic recordings reported evidence for distinct patterns of brain coordinated activity derived from synchronization measures [9]–[11]. The documentation of unique coordination patterns can reveal which brain areas operate with different levels of coordination and when these differences occur so that, perchance, therapeutical interventions may target specific brain areas. These investigations may as well contribute to the diagnosis of autism early in development [12]. However, as a note of caution, one should keep in mind that the notion of functional connectivity in the literature encompasses a wide variety of mathematical techniques applied to different recording modalities, and hence a direct comparison between studies may be misleading.

While connectivity measures are providing important insight into brain function, an area that remains very much under-investigated relates to the detailed analysis of the background, resting nervous system activity. Examination of noise and fluctuations in neurophysiological signals rather than concentrating on averages and magnitudes as is customary, is crucial for a complete understanding of nervous system function and its relation to behavior [13], as sensory stimuli are known to modulate the ongoing neural activity [14]. From a practical perspective, studying ongoing activity is easier than performing cognitive/behavioral tasks in experimental recordings. Here we present an analysis method that allows one not only to determine functional connectivity in a standardized way, but also to reconstruct the spatio-temporal characteristics of the “noise”, i.e. the random input driving the network in the resting state, which is characterized by a minimal presence of, or attention to, sensory stimulation. The approach is based on recent theoretical work from one of our labs [15], [16], showing that spontaneous brain activity can be described as a stochastic system driven by Gaussian noise that may be spatially and temporally correlated. Importantly, we find that not only the functional brain connectivity, but also the spatial pattern of stochastic background activity can serve as reliable discriminators between individuals with autism and control participants. This suggests that our model provides an efficient biomarker for Asperger’s syndrome, and perhaps more generally, for autism spectrum disorders as well as other cognitive phenotypes.

The outline of our results is as follows. We first investigate global differences between the connectivity matrices in the control and ASD groups. In particular, we show that the matrices of each group cluster in a high-dimensional space. We then investigate which specific features of the matrices account for this clustering. Specifically, we show that certain pair-wise interactions are significantly different. Finally, we demonstrate that in addition to having some different functional connections, the brains from control and ASD differ in the spatial distribution of background noise driving the network.

## Results

In the absence of stimulation, the non-linear dynamics of the brain reduces to noise-driven fluctuations around a state of equilibrium, which in realistic neural-mass models of brain dynamics corresponds to a hyperbolic fixed point [15], [17], [18]. The presence of background noise does not allow the system to quench at the fixed point but instead the noise perturbs the system in a continuous manner so that fluctuates around the equilibrium. Thus, consistent with the approach used by several authors [15], [16], [19]–[21], large-scale spontaneous brain activity can be described as a linear multivariate stochastic system, which in its continuous version in time is equivalent to an Ornstein-Uhlenbeck process [22] (see *Methods*)
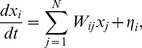
(1)where 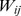 is the functional connectivity matrix, i.e. the coupling between the *j*-th and the *i*-th nodes; 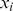 is the neural activity of the *i*-th node with respect to baseline, measured as the signal from the *i*-th MEG channel; 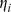 are the residuals (background, uncorrelated white noise) of the *i*-th channel; and *N* is the number of nodes (channels). The sign of *W_ij_* can be thought of as *functional* excitation and inhibition, although these do not necessarily represent excitatory and inhibitory synaptic connections at the cellular level. From a physiological perspective *W_ij_* can be thought of as the net effect of many excitatory and inhibitory synapses plus other neuromodulators converging onto the area associated with a node. The units of *W_ij_* are reciprocal of time, i.e. frequency units.

*W_ij_* can be obtained from the empirical data 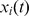 with a linear regression to equation (1). The background noise driving the network 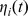 can also be obtained as the residuals of the linear regression. The details of this fitting procedure are provided in *Methods*. The determination of the connectivity matrix and the background noise are the core of our approach and is what allow us to investigate significant differences between the brains of control subjects and those with ASD.

**Figure 1:**
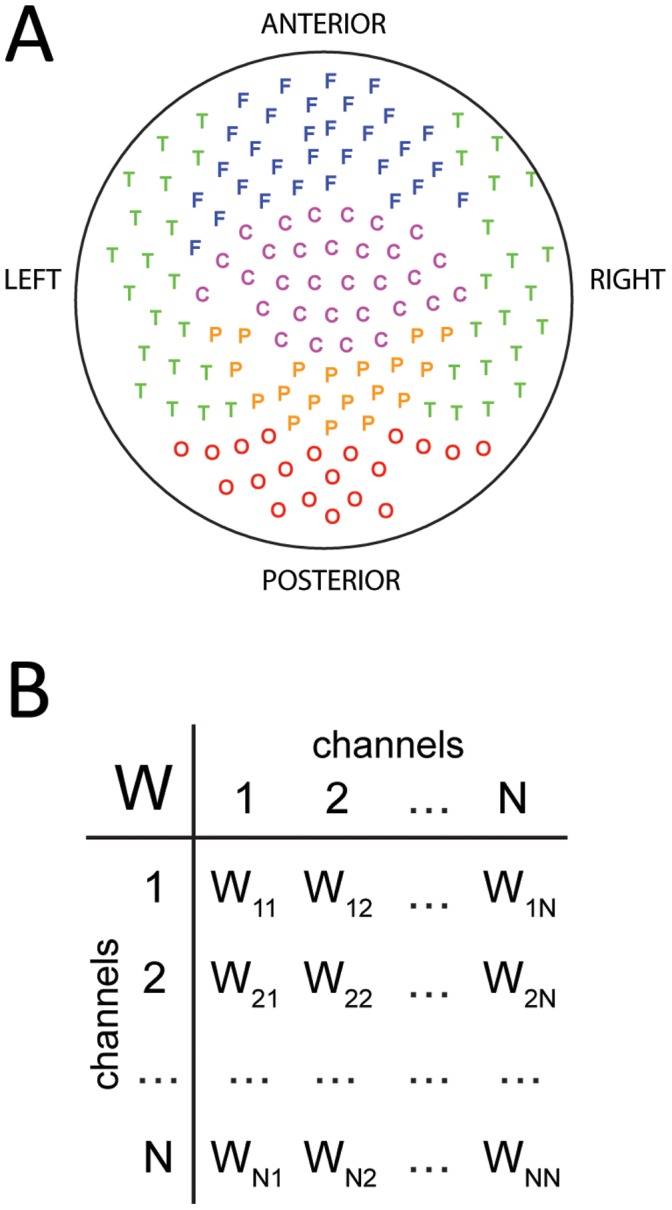
Sensor distribution and functional connectivity. **A)** Stereographic projection of the MEG sensors. The sensor grid covers frontal (F), parietal (P), central (C), temporal (T) and occipital (O) areas from both hemispheres. **B)** Each sensor provides a recording channel. The recorded signals are then used to infer the functional connectivity matrix, 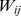 describing the coupling between the areas associated with the *i*-th and *j*-th sensors.

Figure 1a shows a stereographic projection of the MEG sensors distributed over the scalp onto a planar circle. Although the sensor grid has 151 sensors, we only show the positions of the 141 sensors that were used in all the subjects of our study. The other 10 were discarded because they contained artifacts or very low signal-to-noise ratios in at least one subject, thereby precluding their use in comparative analyses. Thus, the dimensions of the functional brain connectivity matrix for each subject are 141×141. The sensors cover the occipital (O), frontal (F), central (C), parietal (P) and temporal (T) areas. Each ordered pair of sensors 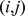 defines an entry in the connectivity matrix *W_ij_* (Fig. 1b) which is obtained from the data using a linear regression to equation (1), as mentioned above.

**Figure 2:**
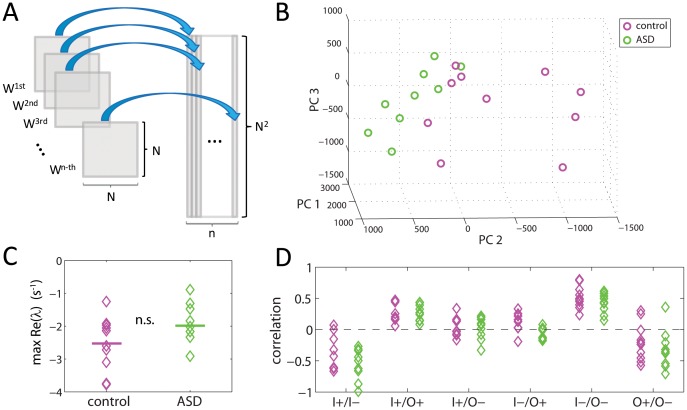
Global properties of the functional connectivity. **A)** Reshaping the connectivity matrices as high-dimensional vectors. **B)** Projection of the vectorized connectivity matrices onto the first three principal components. The matrices of control and ASD are clearly separable. **C)** Linear stability analysis of the connectivity matrices reveals no significant differences between control and ASD. The y-axis plot the largest real part of the eigenvalues of each matrix. **D)** There are no significant differences in the correlation between nodal input and nodal output (I: input; O: ouput; +: excitatory; −: inhibitory).

After obtaining the connectivity matrices for the control subjects (n = 10) and the ASD subjects (n = 9), we investigated if these matrices where significantly different when considered as a whole. To this end, we first “reshaped” the matrices as column vector (Fig. 2A), so that each brain was now represented as a point in a high-dimensional space with 141×141 = 19,881 dimensions. Then, we investigated the separability of the brains from both groups using a support-vector classifier (see *Methods*). The accuracy of the discrimination between both groups was 84% (Table 1). The good separability of the control and ASD groups can be visualized by looking at the projections of the vectors onto their first three principal components, as displayed in Fig. 2B. Note that even in this low-dimensional projection, both groups can be almost perfectly discriminated.

**Table 1:** Separability indices for the connectivity matrices, 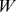.

	Percentage	p-value
**Accuracy**	84	0.0029
**Specificity**	80	0.0780
**Sensitivity**	88	0.0054
**F-Score**	84	0.0013

The fractions of correctly classified individuals were 8/9 for ASD and 8/10 for Control.

Having shown that the functional connectivity matrices for control and ASD subjects are different when considered as a whole, we proceeded to investigate if those differences resulted from difference in the global properties of the matrices. We first investigated the maximal real part of the eigenvalues, which corresponds to a linear stability analysis of system (1). In order for (1) to be a valid model of brain dynamics in the resting state, all eigenvalues must have a negative real part. Otherwise, the brain would be linearly unstable, i.e. epileptic. Figure 2C shows the distribution of the maximal real part of the eigenvalues. There is one data point per matrix corresponding to the eigenvalue with largest real part. The medians of the distributions for the control and ASD cases are not significantly different (p>0.05; Wilcoxon’s ranksum test), indicating that both groups have brains that are equally stable and dissipate perturbations over the same time scale of less than 1 s. We then investigated whether the brains were balanced differently, i.e. whether the distribution of excitation and inhibition across the network nodes was different. A means of keeping a network balanced is by ensuring that each node receives as much excitation as inhibition. This would lead to a high correlation (in absolute value) of the input I+ vector, which is the sum across all rows of the excitatory entries in *W_ij_*, with the I- vector, which is the sum across all rows of the inhibitory entries in *W_ij_*. This is indeed the case for both the control and ASD groups but there are no significant differences between them (Fig. 2D). Another means of keeping the network balanced is by ensuring that each node provides on average as much excitation as inhibition to other nodes in the network. This would lead to a high correlation (in absolute value) of the output O+ vector, which is the sum across all columns of the excitatory entries in *W_ij_*, with the O- vector, which is the sum across all columns of the inhibitory entries in *W_ij_*. That is also the case for both the control and ASD groups but again, there are no significant differences between them (Fig. 2D). Neither are significant differences for the rest of the correlations, I+/O+, I+/O−, I−/O+ and I−/O− (Fig. 2D).

**Figure 3:**
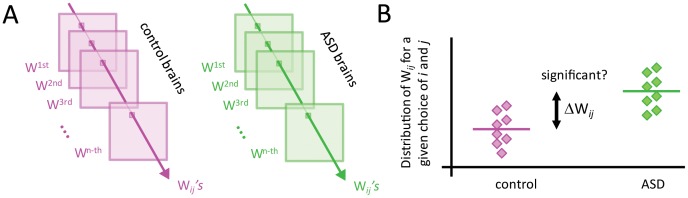
Investigation of pairwise functional connections. **A)** For each pair of channels, 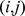 the distribution of values of 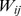 across subjects is built for the control case and ASD. **B)** The comparison of these two distributions allows us to detect significant connectivity changes in ASD relative to control.

**Figure 4:**
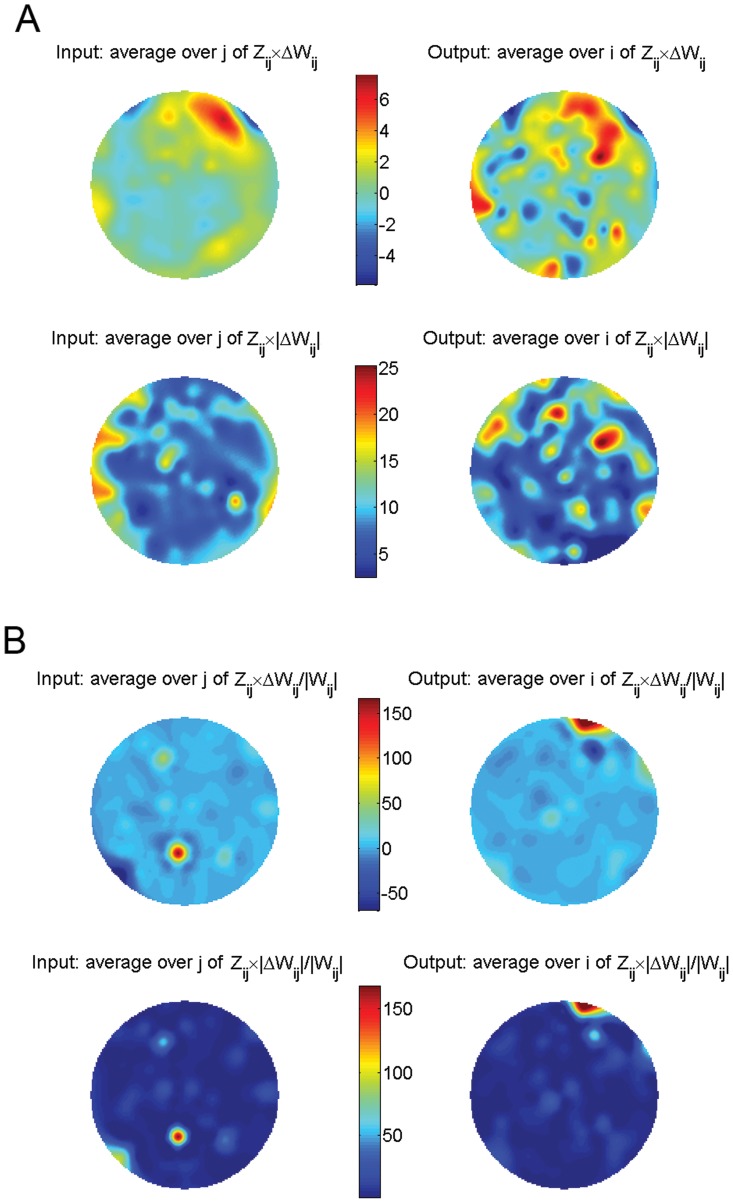
Changes in functional brain connectivity in ASD relative to control. **A)** Changes weighted by their z-score and averaged across inputs or outputs (top) and in absolute value (bottom). **B)** Same as in A) but normalized so that the changes are relative to the connection strength in control.

**Figure 5:**
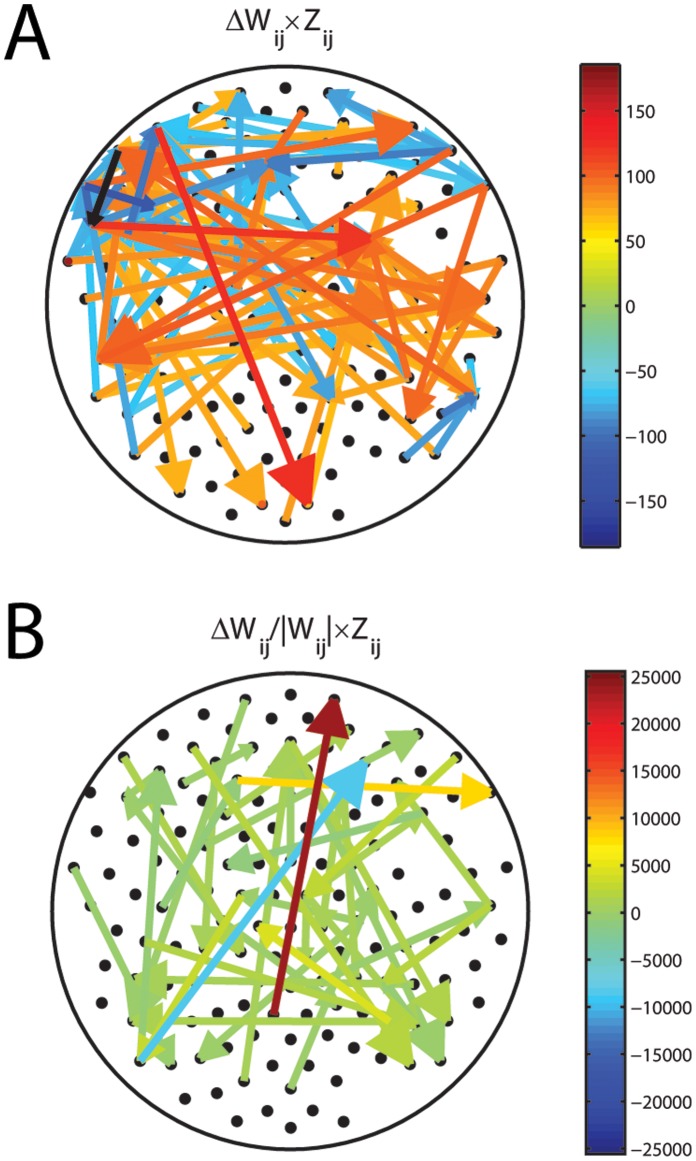
Dominant changes in functional connectivity as an arrow plot. **A)** Absolute changes weighted by their z-score. **B)** Relative changes weighted by their z-score. Clearly, the dominant change in ASD relative to control is an increase in functional excitation from parietal to frontal areas.

Since global properties could not account for the differences between connectivity matrices, we also investigated all pairwise interactions, *W_ij_* and their possible alterations in ASD relative to control (Fig. 3). To this end, for each *W_ij_* we collected all the values across the control and ASD subjects (Fig. 3A) and built the distributions of these values for both groups (Fig. 3B). We then asked if the difference of the means was significant using a random permutation test (see *Methods*). This test also returns the z-score for the actual difference of the means, *Z_ij_*. We recall that a z-score of 1.5 roughly corresponds to a 95% percentile and hence, larger values indicate a highly significant difference. Figure 4A shows the actual difference of the means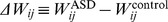, where the *W_ij_* are averaged for each group, weighted by the z-score and averaged across all inputs (left) or outputs (right). The most relevant changes appear in nodes located in frontal, parietal, and temporal areas. Probably more informative is the normalization of these changes relative to the magnitude of the connections in the control case (Fig. 4B), i.e., 
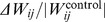
. This representation clearly shows that the major significant change in functional connectivity occurs between parietal and frontal areas. Another way of visualizing these changes is presented in Fig. 5A, for the largest absolute changes, and 5B for the largest relative changes. By far, the increase in functional excitation between a parietal and a frontal area is the largest relative change in connectivity. All together these results demonstrate that there are specific changes in connectivity in ASD relative to control that account for the separability of the brains from these groups when considered as a whole.

**Figure 6:**
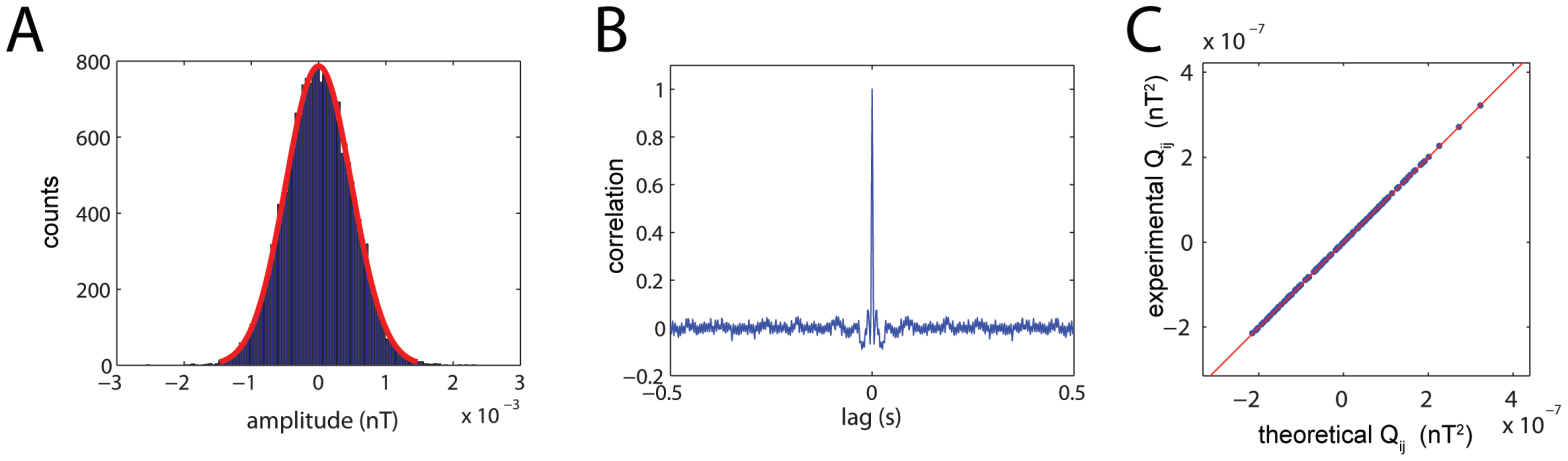
Statistical properties of the background noise driving the brain network. **A)** The noise extracted from each channel is Gaussian. **B)** The noise is not temporally correlated and is practically white. **C)** The covariance of the noise extracted from the data and the covariance of the noise predicted from model (1) match perfectly.

We then asked whether in addition to significant connectivity changes, the background noise driving the brain activity in the resting state could also be different in ASD compared to control. To test this, we obtained the traces of background noise 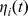
as the residuals from the linear regression of the MEG signals to system (1), as described in *Methods*. We first note that the residuals are normally distributed, as shown in Fig. 6A for the residuals of an arbitrary channel. In addition, the residuals are almost perfectly white, as shown by a sharp centered peak in the autocorrelogram. These results are very important as they provide a validation for model (1). Indeed, if the residuals were neither normally distributed nor white, one would conclude that there is some structure in the data, e.g. a temporal modulation, which cannot be accounted for by a multivariate stochastic linear model. An additional validation for model (1) is provided by the excellent agreement between the covariance matrix of the residuals, *Q_ij_*, as obtained directly from the residuals, and the *Q_ij_* obtained analytically from the connectivity matrix, as explained in *Methods*. This agreement is shown in Fig. 6C for an arbitrary subject as a perfect correlation between the theoretical and experimental values. Altogether, these results demonstrate that model (1) is more than an appropriate and convenient parametric description of the brain dynamics in the resting state: it *is* the actual form of these dynamics.

The matrices 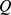 from control and ASD can be well discriminated with a support-vector classifier, as was the case with the connectivity matrices, 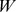 above. Interestingly, the discrimination based on the covariance matrix of the noise is even better: 94% accuracy (Table 2) compared to the 84% accuracy based on the connectivity matrices (Table 1).

**Table 2:** Separability indices for the covariance matrices of the noise, 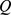.

	Percentage	p-value
**Accuracy**	94	0.0002
**Specificity**	100	0.0044
**Sensitivity**	88	0.0174
**F-Score**	94	0.0002

The fractions of correctly classified individuals were 8/9 for ASD and 10/10 for Control.

**Figure 7:**
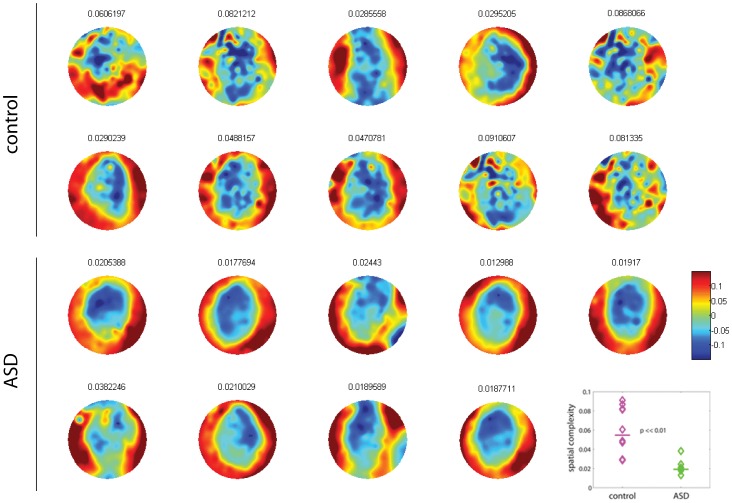
Dominant patterns of background noise driving the brain network. The spatial complexity of the background noise in control is significantly higher than in ASD, as quantified in the inset (Wilcoxon ranksum text, p<<0.01). Numbers on top of the plots show the values of spatial complexity.

The determination of the background noise traces allows us to investigate its spatial structure as well. Indeed, whereas the noise is not temporally correlated (white), it displays spatial correlations. The spatial patterns of the correlated noise are evident from a principal component analysis of the residuals (see *Methods*). To this end, we first compute the principal components as the eigenvectors of *Q_ij_*. Then we calculate the weighted average of the eigenvectors with their eigenvalue as the weight. The resulting vector is plotted as a spatial pattern to visualize the dominant spatial correlations of the background noise for each brain (Fig. 7). We note that quite generally, temporal MEG sensors are spatially correlated as denoted by a similar coloring of these zones. In control brains, the spatial structure is in general *patchier* than in ASD brains. To quantify these observations, we computed the spatial complexity of these patterns with an algorithm that was recently introduced in the literature [23]. Briefly, this algorithm measures how well 2-dimensional interpolation can predict the value of the pattern at the position of a given sensor, given the values in the surrounding sensors (see *Methods*). The values of spatial complexity are significantly higher in control than in ASD (Fig. 7, inset; Wilcoxon ranksum test, p<<0.01). This demonstrates that spatial correlations are constrained to smaller patches on the brain in control subjects, whereas they extend over wide areas in ASD subjects, in other words, there is more variability in the spatial pattern of the background activity (or noise) in children without autism.

## Discussion

Our study has revealed alterations in brain connectivity and background noise in juvenile ASD patients, more specifically, signs of increased excitation were found from occipital to frontal areas. Perhaps even more interestingly, we found that background noise is spatially correlated over wide areas, that is, its spatial complexity is lower in ASD recordings. The analysis has been performed using a novel analytical method to investigate brain activity by determining two of its most fundamental aspects: the direction of functional connections and the temporal and spatial structure of the stochastic inputs driving cortical networks.

There is an increasing demand for adequate discrimination of patients in a variety of psychiatric syndromes using relatively safe and non-invasive methods such as EEG, MEG or neuroimaging recordings. The analytical techniques involved range from pattern recognition of neuroimaging data, as recently shown to classify patients with attention deficit hyperactivity disorder [24], to complexity measures derived from EEGs [12] and graph theory using MEG data [25]. These methodologies normally rely on quantification of activity, sometimes translated as “functional connectivity”, ignoring a most fundamental aspect of nervous system activity: that of the continuous presence of ongoing, background activity. As denoted by some investigators, sensory inputs act as modulators of the ongoing activity [14], and it is in this “noisy” background where intrinsic aspects of each nervous system can be found. Our analysis has the advantage of describing functional interactions through a deterministic component, the functional connectivity, and through a stochastic component, the background noise. We found even better discrimination using the latter, which according to the aforementioned comments should not come as a surprise. Of singular interest is the fact that the spatial variability of the noise is reduced in the recordings from ASD individuals. It is increasingly recognised that decreased variability in physiological signals is associated with disease [26], [27], and specially a reduction of variability in brain signals seems associated with psychiatric and neurological conditions [8]. Hence, our results support the view that cellular activities in brains should present a certain level of fluctuations in order to process information in the considered normal manners. Indeed, evidence for reduced fluctuations in brain signals associated with poorer behavioural performances has been provided recently [28]. From a practical stance, the evaluation of spontaneous brain activity has been proposed as a biomarker in neuropsychiatric disorders [29] and, from a more academic perspective, fluctuations in neurophysiological activity has been proposed to improve the exploration of the brain’s dynamic repertoire [30].

The main connectivity change in ASD relative to control showing enhanced functional excitation from occipital to frontal areas is an indication of another general characteristic of aberrant brain function that is becoming apparent in current research: enhanced neural excitability seems to underlie neuropsychiatric disorders [8] and has been proposed to be the basis for social dysfunction in general [31]. In this regard, autistic-like symptoms in mutant mice is normalized by improving inhibitory neurotransmission using GABA agonists like clonazepam [32]. Perhaps this tendency to show more excitability underlies the well-known epileptic co-morbidity in autism [33]. Abnormal connectivity in autism has been described mostly based on neuroimaging (metabolic) data [3], [34], as was mentioned in the introductory paragraphs above. In our study we find that fronto-occipital sensors display the major differences between the control and ASD group. Alterations of the frontal cortex have been noted in autism, and particularly an abnormal spatial organization in the microglial-neuronal components [35]. Recent tensor imaging studies have also revealed white matter abnormalities in autism, in particular, a possible atypical lateralization in some white matter tracts of the brain and a possible atypical developmental trajectory of white matter microstructure in persons with ASD [36]. Because our measures are derived from MEG signals, and thus detect local population activity mostly in the cortex, we speculate that the observed differences reflect a different activity in frontal cortical areas as these receive processed inputs from other cortical regions, specially sensory ones. Activity in sensory cortices is as essential as that of the normally more considered and studied higher-order association areas; for instance, in behaviors as diverse as the discrimination between free and forced actions, it is the activity at the sensors recording primary sensory cortices that best differential both actions [37]. Because of the great importance of visual inputs, it is perhaps not surprising to see an alteration in occipito-frontal signals.

The term “functional connectivity” has been ambiguously employed to date. In some studies functional connectivity is synonymous with covariance, in some others with synchrony or coherence, etc. We propose here a concept of functional connectivity that has three important advantages: 1) Contrary to previous approaches, we do not focus on the analysis of functional connectivity in the context of psycophysical experiments but rather on ongoing, resting-state activity. This facilitates the estimation of functional connections because the activity of the underlying neural networks does not saturate, so the neural interactions can be well resolved. 2) Our method detects the direction of functional connections, i.e. whether area A excites (or inhibits) area B more strongly or vice versa. Other methods have been previously proposed to detect directionality of network interactions: a) Granger causality, b) the imaginary component of the coherency, and c) the coupling function of phase oscillators. However, methods a) and c) are model-dependent, i.e. they make assumptions about the nature of the signals that oftentimes do not apply to EEG/EMG recordings; and method b) is defined in the frequency domain, so its value depends on the frequency components of the signals. This limits its applicability as a measure of connectivity, which one wants to define by means of a number rather than as a function. 3) To our knowledge, our method is the only one to date that allows one to infer the temporal and spatial structure of the stochastic inputs driving the cortical networks in the brain’s resting state. This is quite remarkable, as the classification of controls and ASD is even more accurate considering the spatial covariance of the noise, 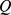 than using the connectivity matrix, 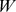, indicating that noise in the brain is an important feature of the cognitive phenotype.

MEG recordings have some limitations to keep in mind. The signals detected by MEG and the source estimates derived from these signals reflect population-scale levels of activity in large neuronal networks. Every individual neuronal component from which an MEG signal is comprised possesses complex non-linear relationships with its synaptically connected neighbors and surrounding glia. The complexity of these interactions cannot be accessed with precise detail from the level of the MEG signal because it provides measurements that are too coarse to reveal such dependencies. As a result, insights gained from the investigation of MEG data are limited to coarse relationships between large populations of cells rather than the detailed understanding of interactions between individual cells. Moreover, spontaneous activity at any given sensor may contain activity from multiple distributed sources, and conversely, the activity of a single signal source can introduce coordinated changes at multiple sensors (cross-talk), which could lead to spurious interactions among MEG sensors. With these caveats in mind, all of our analyses focused on changes in one group (ASD) relative to the other (control). For example, we do not make any conclusions from the absolute connectivity between areas A and B in the brain, but rather from the change in connectivity between A and B in ASD compared to control.

To investigate the cross-talk between sensors we plotted the covariance between two channels as a function of their relative distance in Fig. S1A. The data points are from an individual in the control group but the same pattern is observed in all individuals from both groups. Clearly, for sensors that are less than 10 cm apart, the correlation coefficient between covariance and distance is negative and large in absolute value. There are two components contributing to this negative correlation. One is biological, meaning that anatomical connections are much more likely between nearby areas. This is consistent with previous animal studies showing that nearby neurons in the cortex display synchronized activities *in vivo* [38]–[40]. The other is spurious, indicating cross-talk between sensors. These two components are mixed in the MEG setup and cannot be easily resolved. However, we can show that the trend is comparable between all individuals and indistinguishable between both groups, as shown in Fig. S1B. The distribution of correlation coefficients is statistically the same for control and ASD. This implies that as long as one focuses on changes in functional connectivity of one group relative to the other, the effects of any cross-talk between sensors should cancel out, and therefore, the results will not be contaminated by the limitations of the MEG setup.

Potential limitations in the design of the experiments cannot account for the differences between groups uncovered with our method either. To facilitate the participation of the children in the experiments and minimize distraction, they were asked to press a button at will with their right hand a few times during the recording session (30 s for each subject). Button pressing was not significantly different between both groups, as shown in Fig. S2A (p = 1; Wilcoxon sum-rank test). Data preprocessing, in particular, the removal of a few principal components (PC) from the recordings to filter out eye-blinking and movement artifacts (see *Methods*) did not have differential effects either. Specifically, the number of removed principal components was not significantly different, as shown in Fig. S2 (p = 0.83; Wilcoxon sum-rank test). The gender mismatch is also unlikely to account for differences between both groups. In control there were 6 males and 4 females, whereas in ASD there were 9 males and no females. In this regard we first note, that Asperger’s syndrome is between 4 times [41] and 12 times [42] more frequent in males than females, so it is methodologically very challenging to have sex-matched groups. However, the significant differences that we observe between ASD and control cannot be attributed to a gender-ratio mismatch because the control group is very homogeneous: for example, there are no significant differences in the spatial complexity of the background noise between the boys and girls within the control group (p = 0.76; Wilcoxon rank-sum test). In brief, considering that none of these parameters (button pressing, number of PC removed and gender specificity) were different between groups, it is very unlikely that they can account for the consistent differences in functional connectivity and spatial complexity that we observe between groups.

As in any population study, one must take into consideration the possibility of finite size effects. In statistical terms, the fact that we can establish significant differences in functional connectivity and background noise in relatively small populations suggests that those features are robust. Usually large sample sizes are required to establish the level of significance and accuracy that we obtained in our studies for a smaller sample size. We also note that all the participating children from the ASD group had been clinically diagnosed with Asperger’s syndrome. They clearly had behavioral and cognitive differences with respect to children in the control group. ASD certainly develops over time, but once it is diagnosed based on cognitive parameters it should be possible to observe differences in terms of neural dynamics as well. And that is what we have addressed in this study.

There are two natural extensions of our work for future studies. The first extension is to investigate the applicability of our approach to other cognitive phenotypes to identify alterations in functional brain connectivity and background noise activity. The second extension is methodological and consists in considering nonlinearities in the stochastic model so that it can be applied beyond the resting state to investigate how functional connectivity is modulated by sensory stimulation, attention and other cognitive tasks.

## Methods

### Participants and Magnetoencephalographic Recordings

Data were drawn from a larger sample of children enrolled in a previous study [10]. Data from nineteen children, 9 with Asperger’s syndrome and 10 age-matched control children without any know neurological disorder, were analyzed. Age range was between 6 and 14 years for the controls (mean: 11.2 years; standard deviation: 2.6 years) and between 7 and 16 for ASD (mean: 10.8; standard deviation: 3.5). The 9 children with Asperger’s syndrome were males while the 10 controls were 6 males and 4 females. This gender mismatch is due to the fact that Asperger’s syndrome is between 4 times [41] and 12 times [42] more frequent in males than females, so it is methodologically very difficult to have sex-matched groups (see additional comments on gender mismatch in the *Discussion*). The children's parents provided written consent for the protocol approved by the Hospital for Sick Children Research Ethics Board. Participants met the criteria for ASD based on DSM-IV. Patients were evaluated by the psychologists in the Autism Research Unit of the Hospital for Sick Children or were recruited from the Geneva Centre for Autism and Autism Ontario.

Magnetoencephalographic (MEG) recordings were acquired at 625 Hz sampling rate, DC-100 Hz bandpass, third-order spatial gradient noise cancellation using a CTF Omega 151 channel whole head system (CTF Systems Inc., Port Coquitlam, Canada). Out of the 151 sensors, we discarded 10 that were not comparable across all patients due to artifacts or a very low signal-to-noise ratio. Our analysis thus focused on the recordings from the remaining 141 sensors in all patients. Subjects were tested supine inside the magnetically shielded room. Head movement was tracked by measuring the position of three head coils every 30 ms, located at the nasion, left and right ear, and movements less than 5 mm were considered acceptable. Children were instructed to remain at rest during the recording session that lasted between 30 and 60 s per child. To facilitate the involvement of the children in the experiment and minimize distraction, they were asked to press a button at will with their right hand a few times during the recording session. For each child, an epoch of 30 s was taken off for analysis of functional brain connectivity. All children were awake and had their eyes open during the experiment.

Eye-blinking and muscular artifacts were present in most recordings. These artifacts appeared across many channels with high amplitude relative to baseline fluctuations and thus dominated the first few principal components of the data. Removal of 1 to 6 principal components efficiently eliminated the artifacts without affecting the actual baseline fluctuations.

### Obtaining W from Recordings of Brain Activity

Rewriting system (1) in vector notation one has
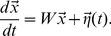
(2)


For a multivariate Ornstein-Uhlenbeck process like system (2) the time-lagged covariance,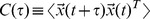, where 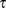
is the lag and the brackets indicate a temporal average, satisfies
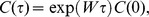
where 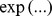 is the exponential matrix function. For 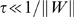, with 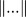 indicating the matrix norm, one has



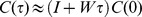
where 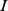
is the identity matrix. This allows us to obtain an expression for the connectivity matrix, 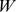
as a function of the lag, 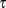




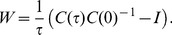
(3)Then, to compute the empirical 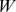, we choose 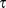
such that the trace of the covariance matrix of the residuals 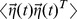
is minimal, which corresponds to minimizing the mean quadratic error of model (2). In our data, this is attained for the smallest possible value of 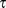 with a sampling frequency of 625 Hz, 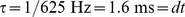, where 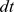
is the integration time step.

Finally, we note that if the zero-lag covariance matrix of the data, 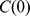, is not invertible, one may replace its inverse matrix, 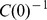
with its pseudo-inverse in equation (3).

### Theoretical and Empirical Q

For a multivariate Ornstein-Uhlenbeck process like (2), the covariance matrix of the residuals, 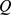
is related to the covariance matrix of the signals, 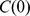 and the drift operator (connectivity matrix, 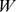) via
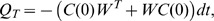
where the subindex *T* refers to a theoretical calculation of 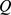. Obviously, the covariance matrix of the residuals can also be directly calculated from the data, which we refer to as empirical estimation with subindex *E*. To this end, one first computes the time derivatives of the signals, 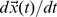
and obtains the residuals as the difference 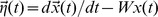. Then, one computes their covariance matrix as



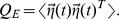



The fact that 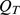 and 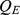
are practically identical, as shown in Fig. 6C, provides a strong validation of model (2).

### Support Vector Classifier

The vectors representing reshaped connectivity matrices (see Fig. 2A) are fed into the algorithm of the support vector classifier [43], [44]. The output of the algorithms returns a set of 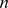 “support vectors”, 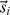, weights 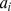, and bias 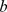
that are used to classify a given reshaped connectivity matrix, 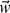, according to the following equation
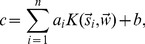
(4)where 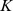
is a *kernel* function. In the case of a linear kernel, which is the one used here, it is the dot product: 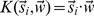 and equation (4) defines a plane in the high-dimensional space of the reshaped matrices. If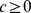, then 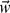 is classified as a member of group 1 (e.g. control), otherwise it is classified as a member group 2 (e.g. ASD). The results of the classification analysis are shown in Table 1. A similar analysis can be performed to classify the reshaped *Q* (see Table 2).

### Group Separability and Cross-validation Techniques

Group separability was addressed by comparing the performance of the support vector classifier with a linear kernel on the original groups to the performance of the same type of classifier on randomized groups (obtained by randomly permuting the group labels) [45]. In our specific implementation, the randomization was carried over 10,000 times. Classification performance was computed for both, the original groups and the randomizations, by leave-one-out cross-validation on every subject. A feature vector for each subject is made using the connectivity matrix *W* (size: 141×141) reshaped into a one-dimensional list of values, a vector of features of dimension 19,881×1. The original Accuracy, Specificity, Sensitivity and F-Score of the classifier were then compared to those obtained for the randomizations. For each one of these four parameters, p-values were obtained by finding the average number of times that each parameter in the randomized population had equal or larger values than those of the classification from the original groups. This test provides a measure of the statistical significance of the classifier performance as well as general group differences. The results are shown in Table 1. The same procedure applied to the covariance matrix of the noise *Q*, produces the results shown in Table 2.

### Random Permutation Test and z-scores

To determine if a given element of the connectivity matrix, 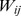
is on average different in ASD from control, we applied the following random permutation test. We took the *n = *10 values from the control group and *m = *9 values from ASD group, as depicted in Fig. 3. To test whether the difference of the means in both groups was significantly different, we first gathered the *n+m* data points into one group and draw *n* points randomly to form a new group, allocating the remaining *m* data points to form a second group. This way we created a random surrogate data set from which the difference of the means was calculated. We then iterated this process 10,000 times to build a probability distribution of the difference of the means for surrogate data. If the difference of the means from the actual data was larger than the 99 percentile of this distribution, then that value was considered to be statistically significant with 99% confidence. The p-value was calculated as the integral of the distribution from left end (−∞) up to the actual value.

We note that the distribution of the difference of the means for the surrogate data converges fairly quickly to a Gaussian as the number of surrogate samples increases. This allows us to easily compute the z-score of the change in connectivity as the actual difference of the means divided by the standard deviation of this distribution.

### Spatial Complexity

Spatial complexity was calculated using a similar algorithm to that already described in [23]. The spatial pattern was obtained as the weighted average of the principal components (eigenvectors of 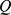) according to their variance (eigenvalue). For each subject this procedure results in a vector *X* of 141 values which is then fed into the spatial complexity algorithm. The intuition behind the algorithm is to capture the heterogeneity of the spatial distribution of values. A pattern is considered to be spatially complex if it contains values at some spatial location (sensor position) that are badly predicted by the values of the neighbouring sensors. The algorithm calculates the squared root of the mean squared difference of each value in *X* with the value predicted at the respective location by a smooth interpolation of its neighbour values using MATLAB griddata v4 method. Higher values of this algorithm correspond to more complex patterns.

## Supporting Information

Figure S1 Quantification of cross-talk between sensors. **A)** Covariance between the signals from a given pair of sensors versus the relative distance between those sensors in a sample subject. Red line displays best linear fit in a short-distance range. The correlation coefficient is given by r. **B)** Distribution of correlation coefficients between covariance and distance for all subjects. Both groups are indistinguishable, meaning that the cross-talk cannot account for differences between groups.
(TIF)

Figure S2 Experimental paradigm and data preprocessing cannot account for differences between groups. **A)** Distributions of button-pressing. **B)** Number of removed principal components (PC).
(TIF)

## References

[1] Herbert MR (2005) Large brains in autism: the challenge of pervasive abnormality. Neuroscientist 11: 417–440. 16151044 10.1177/0091270005278866

[2] Uhlhaas PJ, Singer W (2007) What do disturbances in neural synchrony tell us about autism? Biol Psychiatry 62: 190–191. 17631116 10.1016/j.biopsych.2007.05.023

[3] Rippon G, Brock J, Brown C, Boucher J (2007) Disordered connectivity in the autistic brain: challenges for the “new psychophysiology”. Int J Psychophysiol 63: 164–172. 16820239 10.1016/j.ijpsycho.2006.03.012

[4] Cherkassky VL, Kana RK, Keller TA, Just MA (2006) Functional connectivity in a baseline resting-state network in autism. Neuroreport 17: 1687–1690. 17047454 10.1097/01.wnr.0000239956.45448.4c

[5] Mizuno A, Villalobos ME, Davies MM, Dahl BC, Muller RA (2006) Partially enhanced thalamocortical functional connectivity in autism. Brain Res 1104: 160–174. 16828063 10.1016/j.brainres.2006.05.064

[6] Monk CS, Peltier SJ, Wiggins JL, Weng SJ, Carrasco M, et al (2009) Abnormalities of intrinsic functional connectivity in autism spectrum disorders. Neuroimage 47: 764–772. 19409498 10.1016/j.neuroimage.2009.04.069 PMC2731579

[7] Di Martino A, Kelly C, Grzadzinski R, Zuo XN, Mennes M, et al (2011) Aberrant striatal functional connectivity in children with autism. Biol Psychiatry 69: 847–856. 21195388 10.1016/j.biopsych.2010.10.029 PMC3091619

[8] Perez-Velazquez JL, Frantseva MV (2011) The Brain-Behaviour Continuum: The subtle transition between sanity and insanity: World Scientific Publishing Company.

[9] Murias M, Webb SJ, Greenson J, Dawson G (2007) Resting state cortical connectivity reflected in EEG coherence in individuals with autism. Biol Psychiatry 62: 270–273. 17336944 10.1016/j.biopsych.2006.11.012 PMC2001237

[10] Perez Velazquez JL, Barcelo F, Hung Y, Leshchenko Y, Nenadovic V, et al (2009) Decreased brain coordinated activity in autism spectrum disorders during executive tasks: reduced long-range synchronization in the fronto-parietal networks. Int J Psychophysiol 73: 341–349. 19465065 10.1016/j.ijpsycho.2009.05.009

[11] Teitelbaum A, Belkas J, Brian J, Perez Velazquez JL (2012) Distinct patterns of cortical coordinated activity in autism. In: Richardson CE, Wood RA, editors. Autism Spectrum Disorders: New Research. Hauppauge, New York: Nova Science Publishers.

[12] Bosl W, Tierney A, Tager-Flusberg H, Nelson C (2011) EEG complexity as a biomarker for autism spectrum disorder risk. BMC Med 9: 18. 21342500 10.1186/1741-7015-9-18 PMC3050760

[13] Garrett DD, Kovacevic N, McIntosh AR, Grady CL (2011) The importance of being variable. J Neurosci 31: 4496–4503. 21430150 10.1523/JNEUROSCI.5641-10.2011 PMC3104038

[14] Buzsaki G (2006) Rhythms of the Brain: Oxford University Press.

[15] Galán RF (2008) On how network architecture determines the dominant patterns of spontaneous neural activity. PLoS ONE 3: e2148. 18478091 10.1371/journal.pone.0002148 PMC2374893

[16] Steinke GK, Galán RF (2011) Brain rhythms reveal a hierarchical network organization. PLoS Comput Biol 7: e1002207. 22022251 10.1371/journal.pcbi.1002207 PMC3192826

[17] Robinson PA, Rennie CJ, Wright JJ, Bourke PD (1998) Steady states and global dynamics of electrical activity in the cerebral cortex. Physical Review E 58: 3557–3571.

[18] Robinson PA, Rennie CJ, Wright JJ, Bahramali H, Gordon E, et al (2001) Prediction of electroencephalographic spectra from neurophysiology. Phys Rev E Stat Nonlin Soft Matter Phys 63: 021903. 11308514 10.1103/PhysRevE.63.021903

[19] Tononi G, Sporns O, Edelman GM (1999) Measures of degeneracy and redundancy in biological networks. ProcNatlAcadSciUSA 96: 3257–3262. 10.1073/pnas.96.6.3257 PMC15929 10077671

[20] Sporns O, Tononi G, Edelman GM (2000) Connectivity and complexity: the relationship between neuroanatomy and brain dynamics. Neural Netw 13: 909–922. 11156201 10.1016/s0893-6080(00)00053-8

[21] Barnett L, Buckley CL, Bullock S (2009) Neural complexity and structural connectivity. Phys Rev E Stat Nonlin Soft Matter Phys 79: 051914. 19518487 10.1103/PhysRevE.79.051914

[22] Gardiner CW (2004) Handbook of stochastic methods for physics, chemistry, and the natural sciences. Berlin: Springer.

[23] Garcia Dominguez L, Guevara Erra R, Wennberg R, Perez Velazquez JL (2008) On the spatial organization of epileptiform activity. Chaos 18: 429–439.

[24] Cheng W, Ji X, Zhang J, Feng J (2012) Individual classification of ADHD patients by integrating multiscale neuroimaging markers and advanced pattern recognition techniques. Front Syst Neurosci 6: 58. 22888314 10.3389/fnsys.2012.00058 PMC3412279

[25] Tsiaras V, Simos PG, Rezaie R, Sheth BR, Garyfallidis E, et al (2011) Extracting biomarkers of autism from MEG resting-state functional connectivity networks. Comput Biol Med 41: 1166–1177. 21592470 10.1016/j.compbiomed.2011.04.004

[26] Vaillancourt DE, Newell KM (2002) Changing complexity in human behavior and physiology through aging and disease. Neurobiol Aging 23: 1–11. 11755010 10.1016/s0197-4580(01)00247-0

[27] Buchman TG (2004) Nonlinear dynamics, complex systems, and the pathobiology of critical illness. Curr Opin Crit Care 10: 378–382. 15385755 10.1097/01.ccx.0000139369.65817.b6

[28] McIntosh AR, Kovacevic N, Itier RJ (2008) Increased brain signal variability accompanies lower behavioral variability in development. PLoS Comput Biol 4: e1000106. 18604265 10.1371/journal.pcbi.1000106 PMC2429973

[29] Zhou Y, Wang K, Liu Y, Song M, Song SW, et al (2010) Spontaneous brain activity observed with functional magnetic resonance imaging as a potential biomarker in neuropsychiatric disorders. Cogn Neurodyn 4: 275–294. 22132039 10.1007/s11571-010-9126-9 PMC2974101

[30] Ghosh A, Rho Y, McIntosh AR, Kotter R, Jirsa VK (2008) Noise during rest enables the exploration of the brain’s dynamic repertoire. PLoS Comput Biol 4: e1000196. 18846206 10.1371/journal.pcbi.1000196 PMC2551736

[31] Yizhar O, Fenno LE, Prigge M, Schneider F, Davidson TJ, et al (2011) Neocortical excitation/inhibition balance in information processing and social dysfunction. Nature 477: 171–178. 21796121 10.1038/nature10360 PMC4155501

[32] Han S, Tai C, Westenbroek RE, Yu FH, Cheah CS, et al.. (2012) Autistic-like behaviour in Scn1a(+/−) mice and rescue by enhanced GABA-mediated neurotransmission. Nature. 10.1038/nature11356 PMC3448848 22914087

[33] Kohane IS, McMurry A, Weber G, MacFadden D, Rappaport L, et al (2012) The co-morbidity burden of children and young adults with autism spectrum disorders. PLoS One 7: e33224. 22511918 10.1371/journal.pone.0033224 PMC3325235

[34] Belmonte MK, Allen G, Beckel-Mitchener A, Boulanger LM, Carper RA, et al (2004) Autism and abnormal development of brain connectivity. J Neurosci 24: 9228–9231. 15496656 10.1523/JNEUROSCI.3340-04.2004 PMC6730085

[35] Morgan JT, Chana G, Abramson I, Semendeferi K, Courchesne E, et al (2012) Abnormal microglial-neuronal spatial organization in the dorsolateral prefrontal cortex in autism. Brain Res 1456: 72–81. 22516109 10.1016/j.brainres.2012.03.036

[36] Travers BG, Adluru N, Ennis C, Tromp do PM, Destiche D, et al (2012) Diffusion tensor imaging in autism spectrum disorder: a review. Autism Res 5: 289–313. 22786754 10.1002/aur.1243 PMC3474893

[37] Kostelecki W, Mei Y, Garcia Dominguez L, Perez Velazquez JL (2012) Patterns of brain activity distinguishing free and forced actions: contribution from sensory cortices. Frontiers in Integrative Neuroscience: in press. 10.3389/fnint.2012.00084 PMC3459011 23060760

[38] Cardoso de Oliveira S, Thiele A, Hoffmann KP (1997) Synchronization of neuronal activity during stimulus expectation in a direction discrimination task. J Neurosci 17: 9248–9260. 9364071 10.1523/JNEUROSCI.17-23-09248.1997 PMC6573624

[39] Constantinidis C, Franowicz MN, Goldman-Rakic PS (2001) Coding specificity in cortical microcircuits: a multiple-electrode analysis of primate prefrontal cortex. J Neurosci 21: 3646–3655. 11331394 10.1523/JNEUROSCI.21-10-03646.2001 PMC6762477

[40] Komiyama T, Sato TR, O’Connor DH, Zhang YX, Huber D, et al (2010) Learning-related fine-scale specificity imaged in motor cortex circuits of behaving mice. Nature 464: 1182–1186. 20376005 10.1038/nature08897

[41] Mattila ML, Kielinen M, Jussila K, Linna SL, Bloigu R, et al (2007) An epidemiological and diagnostic study of Asperger syndrome according to four sets of diagnostic criteria. J Am Acad Child Adolesc Psychiatry 46: 636–646. 17450055 10.1097/chi.0b013e318033ff42

[42] Whiteley P, Todd L, Carr K, Shattock P (2010) Gender Ratios in Autism, Asperger Syndrome and Autism Spectrum Disorder. Autism Insights 2: 17–24.

[43] Boser BE, Guyon IM, Vapnik VN (1992) A training algorithm for optimal margin classifiers. ACM New York.

[44] Vapnik V (1998) Statistical learning theory. New York: Wiley.

[45] Camargo A, Azuaje F, Wang H, Zheng H (2008) Permutation - based statistical tests for multiple hypotheses. Source Code Biol Med 3: 15. 18939983 10.1186/1751-0473-3-15 PMC2611984

