# Supplementary figures and images for: A Model of Functional Brain Connectivity and Background Noise as a Biomarker for Cognitive Phenotypes: Application to Autism
Source: PLoS One. 2013 Apr 17;8(4):e61493. doi: 10.1371/journal.pone.0061493 (PMC3629229; doi:10.1371/journal.pone.0061493)

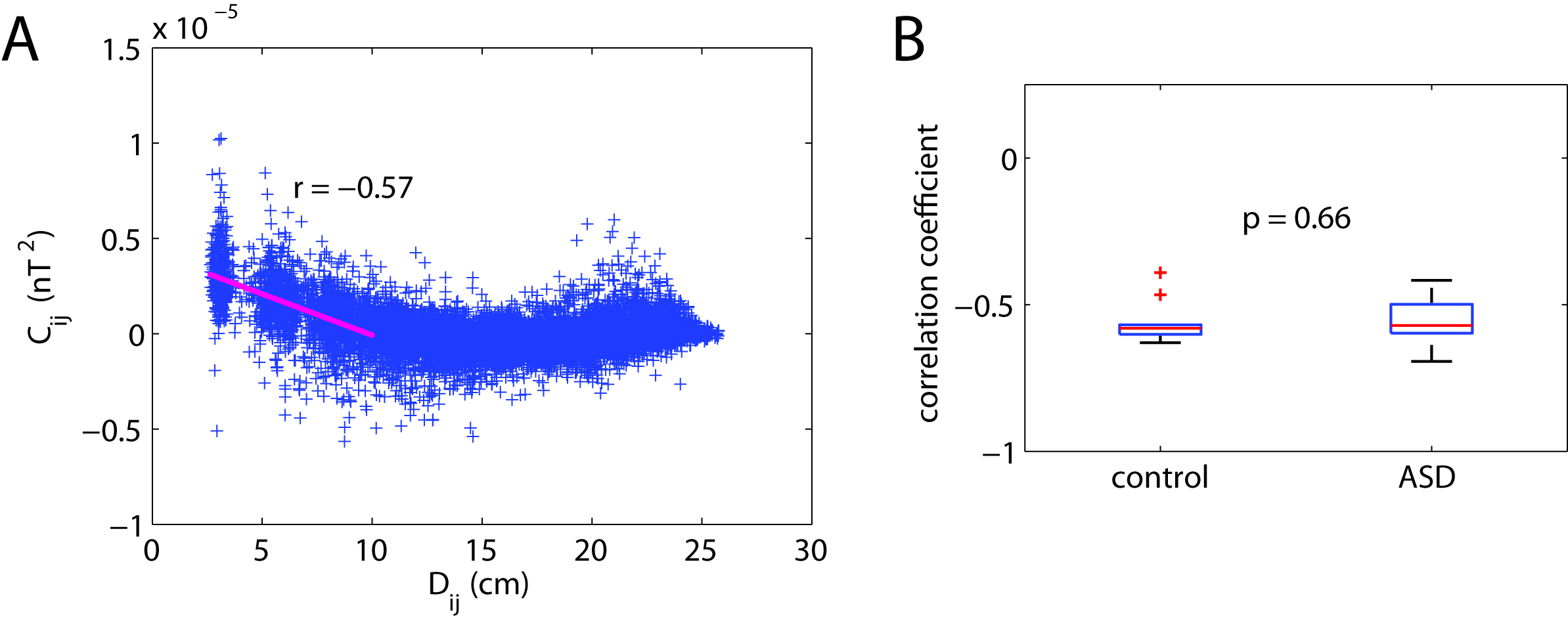

Supplement: Figure S1 — Quantification of cross-talk between sensors. A) Covariance between the signals from a given pair of sensors versus the relative distance between those sensors in a sample subject. Red line displays best linear fit in a short-distance range. The correlation coefficient is given by r. B) Distribution of correlation coefficients between covariance and distance for all subjects. Both groups are indistinguishable, meaning that the cross-talk cannot account for differences between groups. (TIF) [file pone.0061493.s001.tif]

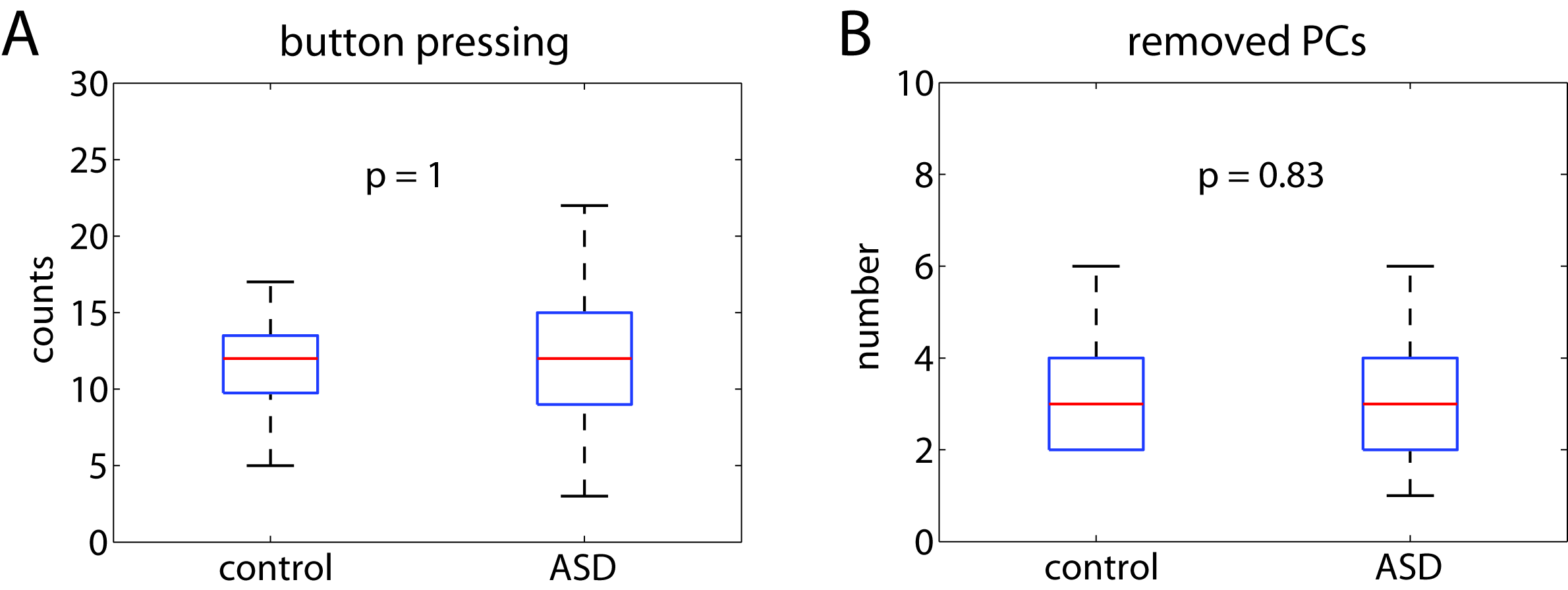

Supplement: Figure S2 — Experimental paradigm and data preprocessing cannot account for differences between groups. A) Distributions of button-pressing. B) Number of removed principal components (PC). (TIF) [file pone.0061493.s002.tif]
